# Precarious working conditions and psychosocial work stress act as a risk factor for symptoms of postpartum depression during maternity leave: results from a longitudinal cohort study

**DOI:** 10.1186/s12889-020-09573-w

**Published:** 2020-10-06

**Authors:** Marlene Karl, Ronja Schaber, Victoria Kress, Marie Kopp, Julia Martini, Kerstin Weidner, Susan Garthus-Niegel

**Affiliations:** 1 Department of Psychotherapy and Psychosomatic Medicine, Faculty of Medicine of the Technische Universität Dresden, Dresden, Germany; 2 Department of Psychiatry and Psychotherapy, Faculty of Medicine of the Technische Universität Dresden, Dresden, Germany; 3 Department of Child Health and Development, Norwegian Institute of Public Health, Oslo, Norway

**Keywords:** Postpartum depressive symptoms, Peripartum health, Maternal mental health, Psychosocial work stress, Effort-reward imbalance, Precarious working conditions, DREAM study

## Abstract

**Background:**

The majority of Western women work during their reproductive years, but past research has often neglected the influence of work-related factors on postpartum mental health. Especially postpartum depression (PPD) is an enormous psychological burden for mothers. Therefore, this study aims to investigate the prospective impact of precarious working conditions and psychosocial work stress during pregnancy (such as work-privacy conflict and effort-reward imbalance at the job) on symptoms of maternal PPD.

**Methods:**

In the prospective-longitudinal cohort study DREAM (**DR**esdner Studie zu **E**lternschaft, **A**rbeit und **M**entaler Gesundheit), *N* = 587 employed women were questioned about their work during pregnancy and their mental health 8 weeks after delivery.

**Results:**

Multiple regression analyses revealed that work-privacy conflict, low reward at work, and precarious working conditions significantly predicted symptoms of PPD, even when controlling for lifetime depression, anxiety, education, parity, and age.

**Conclusion:**

Our results indicate that psychosocial work stress and precarious working conditions have important implications for maternal peripartum mental health. They might act as prospective risk factors for PPD during the period of maternal leave. Hence, future research should focus on preventative measures targeting work life.

## Background

Postpartum depression (PPD) is a common complication during the postpartum period, affecting 17.7% of the global female population [1]. Previous research has identified various predictors and risk factors for PPD such as antenatal depression, anxiety, stressful life events [2, 3], and factors associated with PPD ≤ 1 year postpartum such as income inequality, and working ≥40 h a week [1]. PPD represents a major public health problem [4]. It can have serious adverse consequences for the mothers, increasing the challenge to simultaneously care for the offspring, handle household duties, as well as work duties [5]. Moreover, maternal PPD can have serious consequences for the physical, emotional and cognitive development as well as for the behaviour of the child [6–9]. There has been evidence that in general, maternal work participation has a positive influence on female health [10, 11] and might be a protective factor for symptoms of PPD [12]. However, certain aspects of work seem to increase the risk for depression, such as effort-reward imbalance [13] and work-privacy conflict, especially in non-pregnant populations [14, 15]. Studies investigating pregnant populations however are rare. First studies show that poor quality jobs without security, control, flexibility, or maternal leave are associated with an increased risk of maternal postpartum psychological distress [16]. Moreover, a recent study has shown that high job burden during pregnancy is a risk factor for PPD symptoms [17]. Certain psychosocial work stress, such as higher psychological work demands, lower schedule autonomy, and lower perceived control over work and family [18], higher total workload, and lower job flexibility could be associated with symptoms of PPD. [19] Considering the detrimental effects of PPD, these work-related factors and specific aspects of the work environment need to be investigated further to be able to increase the health and well-being of the whole family. Especially since those factors can be directely targeted by work policies and therefore mitigate the burden of PPD relatively easy.

### The relation between working conditions and postpartum depression

#### Precarious working conditions

Employment conditions and employment approaches have changed over the past decades, but the health correlates of *precarious employment* have not yet been sufficiently investigated [20], especially in women. Since there is no standard definition of *precarious employment*, Vives et al. [20] developed an instrument, the Employment Precariousness Scale EPRES, assessing precarious employment considering the following six dimensions: *temporariness* (contract duration), *disempowerment* (level of negotiation of employment conditions), *vulnerability* (defenselessness to authoritarian treatment), *wages* (low or insufficient; possible economic deprivation), *rights* (entitlement to workplace rights and social security benefits), and *exercise rights* (powerlessness, in practice, to exercise workplace rights) [21]. This construct has already been linked to poor mental health in women [22], but not yet to peripartum mental health. The association seems to follow a dose-response function, i.e., the higher the precarious employment, the higher the prevalence of poor mental health in women (and men) [23]. Additionally, the association of working conditions with mental health seems to be of a complex nature as it has been suggested that precarious working conditions might also have an impact on work-privacy-conflict [24]. Therefore, it seems worthwhile to include both concepts in more in-depth investigations.

#### Psychosocial work stress

##### Work-privacy conflict

The historical gender role of mothers only doing housework and taking care of the children without pursuing a career is no longer representing the majority of mothers [25], leading to mothers having two roles – as caretaker at home and as professional at the workplace. Hence, it is important to consider the reciprocal relationship of these two roles including potentially arising conflicts between them. Work-privacy conflict (WPC), and synonymous terms such as work-family conflict and work-life conflict, are understood as experiences from work that impact experiences in the private/family domain [26]. A growing body of research has investigated possible negative consequences of this spill-over, such as higher prevalence of burnout, depression, anxiety, and absenteeism from work as well as lower life satisfaction, lack of energy, sleep disorders, fatigue, and poorer self-reported health [27–29]. In addition, women with depression often reported to have a very high WPC [14] and a 2-year prospective study in Sweden could show that the increased risk of poor self-rated health influenced by WPC was more pronounced in women than in men [30]. In general, mothers and women seem to be at greater risk of WPC [25], especially when holding a university degree or having a high socio-economic status [14]. Given the diversity of different working environments, it seems likely that other aspects of psychosocial working conditions, such as emotional and quantitative demands at the workplace, are related to WPC [14]. Since pregnant women are facing enormous changes within their personal life, such as a heightened time pressure with being employed and caring for the offspring [31], it seems necessary to investigate the impact of WPC on pregnant women and on PPD [18].

##### Effort-reward imbalance

Another concept to explain the impact of work factors on mental health is the effort-reward imbalance (ERI) model. The model claims that a lack of reciprocity between ‘costs’ and ‘gains’ at the workplace may result in a state of emotional distress which can lead to negative consequences [32]. Findings highlight the association of the ERI with mental health problems such as depression [33], emotional exhaustion [34], and burnout [35, 36]. A recent meta-analysis with eight cohort studies, including 84.963 employees from Europe, Canada, and the US, concludes that ERI is associated with an increased risk for depressive disorders. The following three mechanisms are suggested as explanations: feelings of humiliation and deteriorating self-esteem caused by the mismatch of effort and reward; perception of entrapment and learned helplessness; and a dysregulated hypothalamic-pituitary-adrenal stress (HPA)-axis caused by the mismatch [13]. Although more research is necessary, Siegrist et al. [37] emphazised the idea of biomarkers, such as a dysregulated HPA-axis and altered functions of immune and inflammatory markers, acting as mediators of work stress and stress-related disorders such as depression.

Up to date, only very few studies examined the ERI ratio within peripartum populations using pregnancy-related outcomes, even though this has been suggested in prevoius research on PPD [17]. Lee et al. [38] investigated whether maternal work-related stress during pregnancy influenced birth related outcomes such as birthweight and gestational age. As ERI *reward* scores increased, gestational age also significantly increased. Moreover, an inverse relationship between the ERI *ratio* and gestational age has been observed. In this study, the ERI ratio was not significantly associated with birthweight. In contrast, Meyer, O’Campo, Warren, & Muntaner [39] could demonstrate such an association by examining a sample of 61 women multiple times over the course of their pregnancy. A declining ERI ratio was associated with higher birthweight. The authors argued, that the accumulated disadvantage represented by the ERI ratio might have a negative effect on the health of the mother. The effect on birthweight was robust to the inclusion of other occupational factors and stressors in the regression model such as WPC. In addition, an earlier study found that an increasing ERI ratio was associated with higher systolic blood pressure in the peripartum period [40]. Given this evidence, it was suggested to combine the ERI measure with other work stress identicators, such as WPC to investigate depressive symptomatology [41]. Up to date, no profound investigation on PPD and work-related factors including multiple standardized tests to explore psychosocial work stress and precarious working conditions in expectant mothers has been conducted.

### Aims and objectives

To close this gap, this study will examine working conditions during pregnancy and PPD symptoms at 8 weeks postpartum. Its aim is to investigate the impact of precarious working conditions and psychosocial stress factors at work (WPC and effort-reward imbalance) on symptoms of PPD to detect possible prospective risk factors for PPD in expectant mothers.

## Methods

### Study setting and participants

This investigation is part of the longitudinal cohort Dresden Study on Parenting, Work, and Mental Health (DREAM; **DR**esdner Studie zu **E**lternschaft, **A**rbeit und **M**entaler Gesundheit), which prospectively examines the relationship between parental work participation, role distribution, stress factors, and their effects on perinatal outcomes and long-term family mental and somatic health [42]. Expectant mothers and their partners were recruited during pregnancy predominately at information evenings in hospitals and birth preparation courses in and around Dresden, Germany. The DREAM study consists of currently six measurement points. Participants complete questionnaires covering a comprehensive field of physical and mental health outcomes. The measurement points encompass T1, during pregnancy, and five postpartum assessment waves: T2 at 8 weeks, T3 at 14 months, T4 at 2 years, T5 at 3 years and T6 at 4.5 years after birth, for a detailed description see Kress et al [42]. For the purpose of the present study, data from expectant mothers on working conditions during pregnancy (T1) and PPD symptoms at 8 weeks postpartum (T2) were analyzed.

### Study population and retention rate

By April 17th 2019, *n =* 1067 expectant mothers had returned the first questionnaire (T1). Of *n* = 967 mothers, who had already received the second questionnaire (T2), *n* = 814 (84.2%) had returned the completed questionnaire (for flow chart see Additional file 1). Study data are collected and managed using Research Electronic Data Capture (REDCap), hosted at the “Koordinierungszentrum für Klinische Studien” at the Faculty of Medicine of the Technische Universität Dresden, Germany [43, 44].

Women were included in the analysis if they either worked work full-time, part-time, in minor employment, were in apprenticeship, or were already on maternal leave or employment ban from a previous job. Female participants on maternal leave or employment ban were asked to answer the questions according to their last 6 months of employment. Women in self-employment were excluded from the analysis as the used measures for work stress include questions about the behavior of supervisors.

All participants were asked about their lifetime history of major depression [45] and *n* = 85 (10.9%) participants responded positively to the symptoms for a lifetime diagnosis, while data for *n* = 35 (4.3%) participants were missing. To assess only incident depression, these participants were excluded from all analyses. Because of missing data and exclusion of outliers, *n* varied slightly between the different analyses.

### Measures

#### Adverse psychosocial work conditions as predictors for PPD

Psychosocial work characteristics were assessed by the EPRES [21], the German version of the ERI [32, 46] and the WPC [47, 48] derived from the Copenhagen Psychosocial Questionnaire (COPSOQ; 49).

The EPRES assesses precarious working conditions and was specifically designed for epidemiological studies among waged workers [20]. In previous investigations, it has shown good acceptability, internal consistency, reliability (Cronbach’s alpha coefficients ≥0.70), and construct validity [20]. The subscale *disempowerment* was excluded in this study due to many different types of employment within the sample. The overall EPRES score, ranging from 0 (not precarious) to 4 (most precarious), is the arithmetic mean of all subscale scores.

The COPSOQ was designed to assess a range of psychosocial factors at the workplace [49]. For the purpose of this study, its subscale WPC [47, 48] with 7 items from the German version of the COPSOQ was used. The WPC has been associated with overall demands at work, specifically with cognitive and emotional demands and has been previously shown to be associated with depression [14]. The scale has very good internal consistency (Cronbach’s alpha = 0.92;49). Response categories ranged from “to a small extent” (1) to “to a large extent” (5), and were transformed to fit the range 0–100 [49]. High levels on the subscale indicate a higher WPC, meaning that work interferes with a person’s private life.

The ERI displays the psychosocial working conditions and a possible imbalance between effort put into the job and reward achieved from it. The standardized self-report measure consists of three subscales: *effort, reward*, and *overcommitment*. For the purpose of this study, the short-version of the ERI was used [32]. It includes three items concerning *effort* and seven items concerning *reward.* The ERI has satisfactory validity [32] and the short version has been applied in German industrial workers with good psychometric properties (overall Cronbach’s alpha ≥0.77; [50]). Response categories ranged from “full disagreement” to “full agreement with the respective statement”. Sum scores were calculated for the two subscales, where higher scores reflect higher effort and higher reward. The ERI ratio, the core indicator of the ERI model, was then calculated by dividing the *effort* by the *reward* subscale sum scores, weighed by the number of items [51]. Higher values of the ratio express a higher level of imbalance between (high) effort and (low) reward.

#### Outcome measure for symptoms of postpartum depression

PPD was measured by the German version of the Edinburgh Postnatal Depression Scale (EPDS; [52]). The EPDS is the most common scale to screen for symptoms of PPD across the perinatal period and has been validated in numerous studies [53]. The EPDS is a 10-item self-report scale, scored on a four point scale (0–3; [52]). It ranges from 0 to 30. Higher scores indicate stronger symptoms of PPD. For the sample description, the prevalence of PPD will be reported using the most frequently used cut off score of ≥10 to indicate minor depression [52, 54].

#### Covariates

Age, parity, anxiety, and professional education were selected as potential confounders. Younger age, anxiety, and low education might be a risk factor for PPD [2] and might be associated with the perception of a less stable/favorable employment. Parity, as a risk factor for PPD, also has an impact on female employment [55] and was therefore considered as a covariate (no child / 1 or more children). Anxiety was measured with the anxiety subscale of the validated German version of the Symptom-Checklist Revised (SCL-90-R; [56]). The scale ranges from 0 to 40 and higher scores indicate higher levels of anxiety [56].

### Statistical analyses

Descriptive and correlational analyses were carried out to acquire information on the prevalence and link between symptoms of PPD, psychosocial work stress, and precarious working conditions within the sample. Possible missing values for items of a sum score were substituted with the participant’s mean value if no more than 20% of items were missing on this scale. Before the analysis, outliers were excluded and analysis of assumptions, including multicollinearity, was conducted according to Field [57]. Thereafter, linear regression models were individually calculated to analyze the prospective influence of precarious working conditions and the psychosocial work stress factors on symptoms of PPD (used as a continuous measure). Due to the prospective design, possible risk factors as defined by Kraemer et al. [58] could be identified. To investigate whether the predictors have an individual impact on the outcome, the predictors were separately tested for their prospective impact on PPD (see Tables 2 and 3) before using all predictors within one regression model to differentiate whether each predictor explain separate parts of the variance in PPD symptoms (see Tables 4 and 5). Forced entry was used. All predictors and covariates showed sufficient internal consistency with Cronbach’s alpha scores > .70 with the exception of the scale *rights* from the EPRES questionnaire (Cronbach’s alpha = .30). All analyses were conducted using IBM SPSS Statistics [59].

## Results

### Sample characteristics

The final sample consisted of *n* = 587 mothers. The sample characteristics are summarized in Table 1.

**Table 1:** Sample characteristics at T1 (during pregnancy) and depressive symptoms at T2

Sample characteristics	*n*^a^ (%)	Mean ± *SD* (Range)
Age		30.10 ± 3.88 (20–43)
Week of pregnancy		30.49 ± 5.94 (12–40)
**Country of birth**		
Germany	571 (97.6)	
Other	14 (2.4)	
**Education**		
Lower secondary education	5 (0.9)	
Secondary school certificate	121 (20.6)	
Advanced technical college entrance qualification	50 (8.5)	
Subject-related or higher education entrance qualification (A-level)	411 (70.0)	
**Professional education**		
No university degree	244 (41.6)	
University degree	342 (58.4)	
**Children**		0.25 ± .525 (0–3)
None	461 (79.1)	
One child	104 (17.8)	
Two	14 (2.4)	
Three	4 (0.7)	
**Partnership status**		
Married/registered same sex partnership	245 (41.8)	
Unmarried	319 (54.4)	
Divorced	20 (3.4)	
Widowed	1 (0.2)	
Unknown	1 (0.2)	
**Employment status**^**b**^		
Full-time employment	286 (48.7)	
Part-time employment	89 (15.2)	
Marginal employment	17 (2.9)	
Still in apprenticeship	4 (0.7)	
Employment ban	176 (30.0)	
Parental leave	21 (3.6)	
**Monthly income of main job (after taxes)**		
Less than 450 €	14 (2.4)	
451–850€	8 (1.4)	
851–1500€	176 (30.3)	
1501–2500 €	325 (56.0)	
**Anxiety** ^**c**^ **(Range 0–40)**		2.30 ± 2.67 (0–22)
**EPDS**^**d**^ **at T2 (Range 0–20)**		5.53 ± 3.65 (0–20)
EPDS ≤9	503 (86.3)	
EPDS 10–12	51 (8.7)	
EPDS ≥13	29 (5.0)	

*Note.* ^*a*^ n slightly varies due to missing data of some participants. ^b^ Multiple answers allowed. ^c^ Subscale of Symptom-Checklist Revised (SCL-90-R). ^d^ *EPDS* Edinburgh Postnatal Depression Scale

With 58.4% of the sample holding a university degree, the sample consisted of women with a higher education compared to the average female population of Dresden [60]. At 8 weeks after the anticipated birth (T2), the infants were between about 8 weeks old (*M* = 8.58, *SD* = 2.42). The average EPDS score was 5.53 (*SD* = 3.65) and 83 women (13.7%) had scores of at least 10, indicating a minor depression [52, 54]. This prevalence is similar to recent findings in Germany regarding PPD [61].

### Dropout analyses

Dropout analyses were conducted for sociodemographic characteristics, predictors, and confounders (tables on request). Significant differences between completers vs. non-completers were only found for the EPRES scores and for the relationship status of completers vs. non-completers. Completers had slightly less precarious working conditions (MW = 1.03 vs. MW = 0.94; t (748) = − 2.44, *p* = .001) and were more often in a permanent relationship (99.2% vs. 97.4%; χ2 (1, *n* = 828) = 3.88, *p* = .049).

### Precarious working conditions and psychosocial work stress

#### Precarious working conditions

Precarious working conditions measured with the overall EPRES score (*M* = 1.02, *SD* = 0.46) were significantly positively correlated with PPD (*r* (528) *=* .209, *p* < .001), with *r* indicating a small to medium effect size. Moreover, the EPRES score were significantly positively associated with symptoms of PPD (see Table 2, Model 1) when controlling for anxiety, age, parity, and professional education at T1 (*β* = .157, *p* < .05). EPRES scores were slightly lower in comparison to a healthy sample in Sweden [62]. To better differentiate between different aspects of precarious working conditions a regression model with the EPRES subscales was conducted (see Table 2, Model 2). Only the subscale *wages* showed a significant positive association with symptoms of PPD (*β* = .158, *p* < .05), whereas the other subscales showed no significant association. Since higher wages are associated with a university degree [63] two explorative multiple linear regressions were conducted, this time only including participants with a university degree vs. participants without a university degree. This effect of the subscale *wages* having the biggest impact, could especially be shown in participants with a university degree (*β* = .191, *p* < .05), whereas the association could not be found in participants without a university degree (data not shown).

**Table 2:** Effects of precarious working conditions on PPD, controlled for age, professional education, anxiety and parity

	Model 1				Model 2			
	*SE B*	β	*p*	*R*^*2*^	*SE B*	β	*P*	*R*^*2*^
Constant	3.285		.008	.148	3.088		0.15	.161
Education	−0.125	− 0.018	.660		0.117	0.017	.695	
Anxiety^a^	0.423	**0.317**	.000		0.395	**0.296**	.000	
Age	0.003	0.003	.939		0.001	0.001	.984	
Parity	−0.493	− 0.059	.170		−0.684	− 0.082	.063	
EPRES^b^	1.144	**0.157**	.000					
EPRES^b^ temporariness					0.018	0.006	.887	
EPRES^b^ vulnerability					0.330	0.069	.141	
EPRES^b^ wages					0.565	**0.158**	.001	
EPRES^b^ rights					0.011	0.003	.950	
EPRES^b^ exercise rights					0.238	0.058	.200	

*Note. SE B* Standard error for unstandardized beta, *β* Standardized beta coefficient, *R*^*2*^ Coefficient of determination. ^a^ Subscale of Symptom-Checklist Revised (SCL-90-R). ^b^ Employment Precariousness Scale (EPRES). Significant associations (*p* < .05) are presented in bold

#### Psychosocial work stress

There was a positive association between ERI and PPD. Pearson correlation between ERI and PPD was *r* (564) *=* .178; *p <* .001, with r indicating a small to medium effect size. Regression analyses were carried out for the ERI ratio (see Table 3, Model 1) and the ERI subscales *effort* and *reward* (see Table 3, Model 2); controlled for age, professional education, anxiety, and parity. The ERI ratio (*M* = 1.09, *SD* = 0.37) was significantly positively associated with the EPDS scores (*β* = .112*; p* < .05). ERI scores were comparable with another healthy sample in Germany [50]. Within the ERI (see Table 3, Model 2), only the subscale *reward* (*M* = 19.21, *SD* = 3.60) was significantly negatively associated with symptoms of PPD (*β* = −.143, *p* < .05).

**Table 3:** Effects of psychosocial work stress on PPD, controlled for age, professional education, anxiety, and parity

	Model 1				Model 2				Model 3			
	*SE B*	β	*p*	*R*^*2*^	*SE B*	β	*p*	*R*^*2*^	*SE B*	β	*p*	*R*^*2*^
Constant	3.214		.005	.123	6.576		.000	.132	3.789		.001	.125
Education	−0.251	−0.037	.369		−0.204	−0.030	.465		−0.255	− 0.037	.361	
Anxiety^a^	0.396	**0.293**	.000		0.393	**0.291**	.000		0.394	**0.286**	.000	
Age	0.012	0.014	.739		−0.010	0.011	.792		0.006	0.006	.880	
Parity	−0.566	−0.068	.108		−0.591	−0.070	.092		−0.427	−0.050	.224	
ERI^b^ ratio	1.018	**0.112**	.007									
ERI^b^ reward					−0.134	**−0.143**	.001					
ERI^b^ effort					0.046	0.026	.523					
WPC^c^									0.026	**0.145**	.000	

*Note. SE B* Standard error for unstandardized beta, *β* Standardized beta coefficient, *R*^*2*^ Coefficient of determination. ^a^ Subscale of Symptom-Checklist Revised (SCL-90-R). ^b^ Effort-reward imbalance (ERI). ^c^ work-privacy conflict (WPC). Significant associations (*p* < .05) are presented in bold

There was also a positive association between WPC and PPD. Pearson correlation between WPC and PPD were *r* (576) = .198; *p <* .001, with r indicating a small to medium effect size. Regression analyses were carried out for WPC (see Table 3, Model 3) controlled for age, professional education, anxiety, and parity. WPC scores (*M* = 30.17, *SD* = 19.26) were significantly positively associated with the EPDS scores (*β* = .145*; p* < .05) but were considerable less than a sample of health care professionals [64].

#### Precarious working conditions and psychosocial work stress

All three predictors correlated with each other, according to Cohens conventions [65], within the range of a medium effect sizes of: *r* (518) = .288; *p < .*001 between EPRES and WPC; *r* (518) = .320; *p* < .001 between EPRES and ERI; and *r* (518) = .400; *p < .*001 between WPC and ERI. When including those three predictors in a multiple regression analysis with PPD as the outcome and controlling for age, professional education, anxiety, and parity, the EPRES (β *= .*131, *p* < .05) and WPC scores (*β* = .094, *p* < .05) remained significantly positively associated with PPD (see Table 4).

**Table 4:** Effects of psychosocial work stress and precarious working conditions on PPD, controlled for age, professional education, anxiety, and parity; *R*^*2*^ = .153

	*B*	*SE B*	β	95% CI	*p*
Constant	1.817	1.281		[−0.699; 4.334]	.157
Education	−0.246	0.294	−0.036	[− 0.823; 0.331]	.402
Anxiety^a^	0.378	0.057	**0.281**	[0.266; 0.490]	.000
Age	0.042	0.040	0.047	[−0.037; 0.121]	.299
Parity	−0.819	0.367	−0.026	[−1.540; − 0.098]	.061
EPRES^b^	0.983	0.343	**0.131**	[.309; 1.657]	.004
WPC^c^	0.017	0.008	**0.094**	[.001; 0.032]	.041
ERI Ratio^d^	0.227	0.430	0.025	[−0.618; 1.072]	.598

*Note. B* Unstandardized regression coefficient, *SE B* Standard error for unstandardized beta, *β* Standardized beta coefficient, *R*^*2*^ Coefficient of determination. ^a^ Subscale of Symptom-Checklist Revised (SCL-90-R). ^b^ Employment Precariousness Scale (EPRES). ^c^ work-privacy conflict (WPC). ^d^ Effort-reward imbalance (ERI). Significant associations (*p* < .05) are presented in bold

Since the *effort* subscale of ERI was not significantly associated with symptoms of PPD, the analysis was replicated with solely the *reward* subscale. In this analysis, all three predictors were variable risk factors for PPD (see Table 5).

**Table 5:** Effects of psychosocial work stress and precarious working conditions on PPD, controlled for age, professional education, anxiety, and parity; *R*^*2*^ = .159

	*B*	*SE B*	β	95% CI	*p*
Constant	4.319	1.758		[0.865; 7.774]	.014
Education	− 0.238	0.292	− 0.035	[− 0.811; 0.335]	.415
Anxiety	0.374	0.056	**0.278**	[0.263; 0.485]	.000
Age	0.032	0.040	0.036	[−0.047; 0.111]	.431
Parity	−0.820	0.365	**−0.097**	[−1.538; − 0.102]	.025
EPRES	0.724	0.365	**0.097**	[0.006; 1.441]	.048
WPC	0.017	0.008	**0.094**	[0.002; 0.032]	.030
ERI Reward	−0.088	0.044	**−0.092**	[−0.175; − 0.001]	.047

*Note. B* Unstandardized regression coefficient, *SE B* Standard error for unstandardized beta, *β* Standardized beta coefficient, *R*^*2*^ Coefficient of determination. ^a^ Subscale of Symptom-Checklist Revised (SCL-90-R). ^b^ Employment Precariousness Scale (EPRES). ^c^ work-privacy conflict (WPC). ^d^ Effort-reward imbalance (ERI). Significant associations (*p* < .05) are presented in bold

## Discussion

The aim of this study was to investigate the prospective influence of work-related factors on symptoms of PPD, i.e., whether precarious working conditions, WPC, and ERI ratio increase the risk for PPD. In this prospective cohort study precarious working conditions, WPC, and ERI ratio were individually significantly positively associated with symptoms of PPD within regression models, when controlling for age, professional education, parity, and anxiety during pregnancy. Within a regression including all three predictors, i.e., precarious working conditions, WPC, and the ERI *reward* scale), all predictors remained significantly associated with PPD. Therefore, precarious working conditions and WPC might act as prospective risk factors for PPD, whereas *reward at work* might act as a protective factor. These findings should be discussed in the light of previously conducted research. Studies, which found a decreased risk for symptoms of depression and employment have often included the general employment status (e.g. employed vs. unemployed) [66, 67] supporting the general benefits of employment for peripartum mental health. However, the present study could differentiate between different work factors that might cause depression.

### Precarious working conditions

Precarious working conditions, especiallylow or insufficient wages might act as prospective risk factors of PPD. The effects seem to spill over into the postpartum period of maternity leave. In the present study, the subscale *wages* showed the strongest association with symptoms of PPD within participants with higher education. The association of *wages* with symptoms of PPD was surprising as a recent meta-analysis found no association between education or income as a risk factor for PPD [68]. On the other hand, some research has indicated that low socioeconomic status is associated with depressive symptoms [69, 70], whereas others found no association between income and PPD but could differentiate between the type of job held and the risk for PPD [71]. A higher association with *vulnerability* was expected since its previously found strong relationship with mental health [20].

### Psychosocial work stress- work-privacy conflict and effort-reward imbalance

A perceived interference from work into private life might act as a prospective risk factor for symptoms of PPD. Moreover, a perceived imbalance between effort put into and reward received from work might act as a prospective risk factor for symptoms of PPD. The effects seem to spill over into the postpartum period. In another a previous study among hospital employees in Switzerland, both concepts were found to be significantly associated with burnout, but WPC was found to be a stronger predictor for burnout than ERI among health professionals [72]. Interestingly, when looking at hospital staff with different levels of professional education, ERI has been found to be more relevant for burnout in tertiary-educated staff. This indicates that in higher educated, professional groups such as therapists, physicians, and medical-technical staff effort-reward imbalance seems to be important for mental health [72]. This could be due to a different perspective on the job such as a higher personal commitment and will to work after hours. Similarly, higher educated professionals generally show higher scores for the subscale ERI effort [51] and therefore might be particularly susceptible to burnout or other negative health outcomes. Hence, future analysis should classify between different kinds of professional groups within the peripartum population to investigate different influences of WPC and ERI on PPD. Moreover, among physicians in a hospital setting, Hämmig [35] could show that exposure to stress and the outcomes burnout and intention to leave the profession were partly or largely mediated by WPC and ERI. Whereas WPC predicted burnout symptoms, the ERI ratio showed the strongest effect for predicting thoughts of leaving the profession, indicating that WPC and ERI measure different aspects of psychosocial work stress and therefore might predict different outcomes [35]. More research is required to draw better conclusions and to develop a more integrated model of psychosocial work stress, its biological foundations, and their interaction with the individual.

Whereas the ERI ratio did not remain a significant predictor of PPD symptoms in a regression with WPC and precarious working conditions (see Table 4), the ERI subscale *reward* did proof to be a significant predictor of PPD symptoms. The *reward* subscale had a stronger association with the outcome in the peripartum period. This has also been found in another study, where *reward,* rather than *effort,* was found to be positively associated with gestational age [38]. A recent review has also claimed that a “crisis of gratification”, where expected and legitimate reward is not being experienced, is very relevant to depressive disorders. Seven epidemiological studies concerning the ERI ratio and depression revealed a two-fold elevated relative risk of incident depressive disorder in Europe with findings pointing towards an altered immune function and inflammatory processes [73].

Concerning the peripartum period. Recent findings indicate that ERI scores can fluctuate during the prepartum period [39, 40]. The factor scores for *effort* and *reward* seem to decline during the course of pregnancy, indicating that women put less effort into work and simultaneously receive less reward from it. *Reward* might be especially relevant for expectant mothers, as Siegrist & Li [37] found that aspects of low reward at work seem to be particularly important for biomarkers and therefore physical correlates of health. *Reward* at work should thus be investigated to a greater extent in pregnant populations. It needs to be noted that a steady ERI ratio was not shown for all participants in those previous studies [39, 40]. Therefore, it seems important to consider individual or systematic differences in the trajectory of the ERI.

### Strengths and limitations

This study is the first to include multiple theoretical approaches of work stress within a representative sample of women in the peripartum period. Moreover, no studies were found that previously applied the WPC and the EPRES to the peripartum period. The present investigation is part of a large prospective-longitudinal cohort study covering many fields of interest regarding employment, mental health, and associated factors [42]. Therefore, it will be possible to incorporate further theoretical concepts and factors in future analyses. Examples include paternal mental health as well as hair cortisol levels of fathers, mothers, and their offspring as biological indicators of stress within the DREAM-study. DREAM aims to further investigate the impact of work-related factors on the well-being of all family members.

However, some possible limitations need to be noted. The applications of findings in this study to the general population might be limited as the sample is highly educated in comparison to the general female population of Dresden. However, previous research has shown that self-selection according to sociodemographic variables such as education had little impact on prevalence estimates [74]. Concerning the predictors, the ERI ratio itself might be modified by the peripartum period due to changing perspectives on work [40]. Changes of the ERI score could not be observed in this study, as there was no measurement point before the pregnancy. Within the EPRES, the subscale *rights,* concerning workplace rights, showed a low internal consistency contrary to the findings of the authors [20, 21]. This could be due to the answer categories: “yes”, “no”, “don’t know”. Most participants of the present study were in an employment situation with the workplace right “paid maternity leave”. While most participants were aware of their right to receive paid maternity leave, most were unaware whether they would receive a dismissal wage. When recoding the answer “don’t know” into “missing”, the internal consistency of the scale rose to Cronbach’s alpha = .67. A further limitation is that dropout analyses showed a slightly higher EPRES score for completers vs. non-completers. Women with more precarious working conditions might have been particularly motivated to take part in the study to promote changes within the workplace for pregnant women. However when comparing predictor scores to other healthy samples, participants in this study might experience less precarious working conditions. This could be due to the highly educated sample making it even more important to screen for precarious employment in a less educated sample.

### Implications

Outcomes and consequences of PPD on the affected women, their families, and especially their children are considered to be severe. Longitutinal research including risk and protective factors is necessary to provide the best basis for effective treatment approaches. The present research indicated that work-related factors need to be considered when screening for PPD or during treatment of PPD. Future analyses should include more confounding variables, such as social support, recurrent depression and paternal mental health, especially within the context of a biopsychosocial model of PPD [3]. The DREAM study will be able to include more concepts and possible covariates, as well as hair cortisol concentrations, in future analysis to present a more comprehensive view on peripartum health.

WPC should be lowered, especially considering the changing private situation of women during the perinatal period. The finding that WPC remained significantly associated with PPD when controlling for other psychosocial stress factors at work highlights the importance of introducing strict work guidelines to prevent employees from working at home after working hours. Previous research suggests that various family-specific support systems, such as family friendly organizational policies and climate can reduce WPC [75]. A growing body of research has investigated family-supportive supervisors, i.e., supervisors at work who promote the management of work and non-work responsibilities and acknowledge their employees private life [76, 77]. Facilitating family-supportive supervisors through training might be an effective approach to improve employee work, family, and health outcomes [76]. Additionally, mindfulness and self-monitoring trainings [78] as well as cognitive-behavioural interventions such as coaching sessions [79] have recently been investigated to successfully reduce WPC.

Further, reward at work seems to be a protective factor against symptoms of PPD for expecting mothers. Women might experience less appreciation and reward at work in their peripartum period due to changing physical capacity or the upcoming period of maternal and shifting priorities away from the job. Valuing the efforts spent at work during late pregnancy might protect mothers from PPD symptoms and additionally prepare them for a better re-entry into employment after maternity leave.

Some factors of precarious employment, especially defenselessness to authoritarian treatment and low or insufficient wages, seem to increase the risk for PPD. Workplace policies could be implemented to reduce this risk. Julià, Vives, Tarafa, & Benach [23] suggested a surveillance system to monitor different precarious employment dimensions and identify populations at risk to reduce mental health impact. Moreover, the high association of *wages* with PPD in comparison to the other predictors in this study raises the discussion of equal payment for women in relation to men.

## Conclusion

The aim of the present prospective-longitudinal cohort study was to measure potential work-related risk factors for PPD. Low or insufficient wages and WPC might act as potential risk factors for PPD. Low or insufficient wages and WPC might act as potential risk factors for PPD. Interventions to reduce precarious working conditions, WPC, and ERI might help to reduce the risk for PPD in the postpartum period. Reward at work might act as a potential protective factor against PPD and should be promoted.

## Supplementary information

**Additional file 1.** Flowchart Dropout, Flow chart of the DREAM study. Notes: T1 during pregnancy, T2 8 weeks after anticipated birth date. Data collection is not finished yet (recruitment ongoing).

## Data Availability

The dataset analysed during the current study is not publicly available due to legal and ethical constraints. Public sharing of participant data was not included in the informed consent of the study. The dataset is available from the corresponding author on reasonable request.

## Abbreviations

PPD: Postpartum depression
EPRES: The Employment Precariousness Scale
WPC: Work-privacy conflict
ERI: Effort-reward imbalance
DREAM: Dresden Study on Parenting, Work, and Mental Health (“DResdner Studie zu Elternschaft, Arbeit und Mentaler Gesundheit”)
T1: Measurement point during pregnancy
T2: Measurement point 8 weeks after the anticipated birth
COPSOQ: Copenhagen Psychosocial Questionnaire
EPDS: Edinburgh Postnatal Depression Scale
SCL-90-R: Symptom-Checklist Revised
