# Supplementary material for: Precarious working conditions and psychosocial work stress act as a risk factor for symptoms of postpartum depression during maternity leave: results from a longitudinal cohort study
Source: BMC Public Health. 2020 Oct 6;20:1505. doi: 10.1186/s12889-020-09573-w (PMC7539402; doi:10.1186/s12889-020-09573-w)
Supplement: Supplementary file 1 — Additional file 1. Flowchart Dropout, Flow chart of the DREAM study. Notes: T1 during pregnancy, T2 8 weeks after anticipated birth date. Data collection is not finished yet (recruitment ongoing). [file 12889_2020_9573_MOESM1_ESM.pptx]

## Slide 1
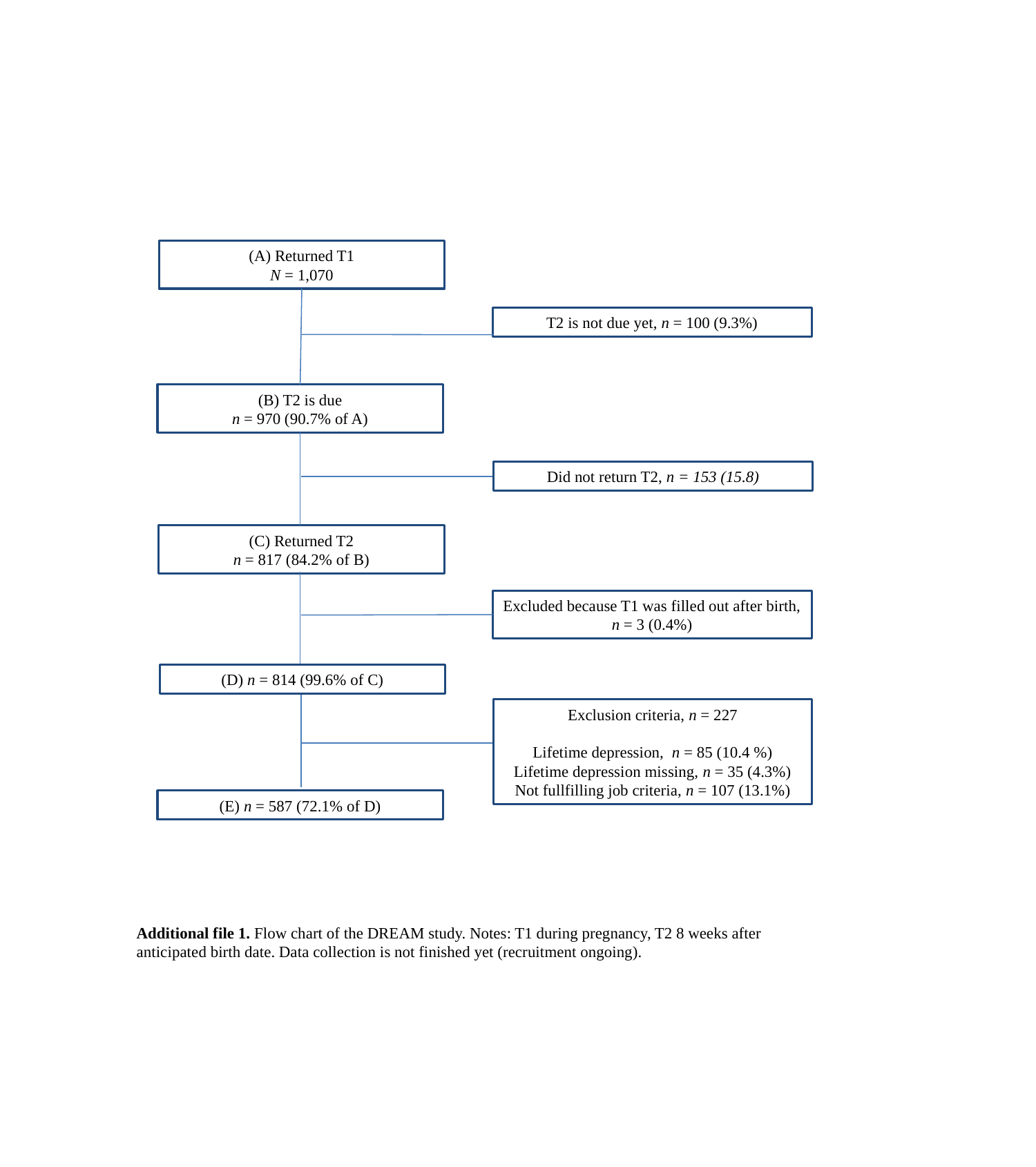

(A) Returned T1
N = 1,070
T2 is not due yet, n = 100 (9.3%)
(B) T2 is due
n = 970 (90.7% of A)
Did not return T2, n = 153 (15.8)
(C) Returned T2
n = 817 (84.2% of B)
Excluded because T1 was filled out after birth, n = 3 (0.4%)
(D) n = 814 (99.6% of C)
Exclusion criteria, n = 227
Lifetime depression, n = 85 (10.4 %)
Lifetime depression missing, n = 35 (4.3%)
Not fullfilling job criteria, n = 107 (13.1%)
(E) n = 587 (72.1% of D)
Additional file 1. Flow chart of the DREAM study. Notes: T1 during pregnancy, T2 8 weeks after anticipated birth date. Data collection is not finished yet (recruitment ongoing).
